# scHDeepInsight: a hierarchical deep learning framework for precise immune cell annotation in single-cell RNA-seq data

**DOI:** 10.1093/bib/bbaf523

**Published:** 2025-10-09

**Authors:** Shangru Jia, Artem Lysenko, Keith A Boroevich, Alok Sharma, Tatsuhiko Tsunoda

**Affiliations:** Laboratory for Medical Science Mathematics, Department of Computational Biology and Medical Sciences, Graduate School of Frontier Sciences, The University of Tokyo, 7-3-1 Hongo, Bunkyo-ku, Tokyo 113-0033, Japan; Laboratory for Medical Science Mathematics, Department of Biological Sciences, School of Science, The University of Tokyo, 7-3-1 Hongo, Bunkyo-ku, Tokyo 113-0033, Japan; RIKEN Center for Integrative Medical Sciences, 1-7-22 Suehiro-cho, Tsurumi, Yokohama 230-0045, Japan; Laboratory for Medical Science Mathematics, Department of Biological Sciences, School of Science, The University of Tokyo, 7-3-1 Hongo, Bunkyo-ku, Tokyo 113-0033, Japan; RIKEN Center for Integrative Medical Sciences, 1-7-22 Suehiro-cho, Tsurumi, Yokohama 230-0045, Japan; Institute for Integrated and Intelligent Systems, Griffith University, 170 Kessels Rd, Nathan, Brisbane, QLD 4111, Australia; College of Informatics, Korea University, 145 Anam-ro, Seongbuk District, Seoul 02841, South Korea; Laboratory for Medical Science Mathematics, Department of Computational Biology and Medical Sciences, Graduate School of Frontier Sciences, The University of Tokyo, 7-3-1 Hongo, Bunkyo-ku, Tokyo 113-0033, Japan; Laboratory for Medical Science Mathematics, Department of Biological Sciences, School of Science, The University of Tokyo, 7-3-1 Hongo, Bunkyo-ku, Tokyo 113-0033, Japan

**Keywords:** cell annotation, cell subtype, single-cell RNA sequencing, deep learning, transformers

## Abstract

Accurate classification of immune cells is crucial for elucidating their diverse roles in health and disease. However, this task remains very challenging in single-cell RNA sequencing (scRNA-seq) data due to the complex and hierarchical relationships of immune cell types. To address this, we introduce scHDeepInsight, a deep learning framework that extends our previous scDeepInsight model by integrating a biologically-informed classification architecture with an adaptive hierarchical focal loss (AHFL). The framework builds on our established method of converting gene expression data into two-dimensional structured images, enabling convolutional neural networks to effectively capture both global and fine-grained transcriptomic features. This design utilizes hierarchical relationships among immune cell types to enhance the classification ability beyond the flat classification approaches. scHDeepInsight dynamically adjusts loss contributions to balance performance across the hierarchy levels. Comprehensive benchmarking across seven diverse tissue datasets shows scHDeepInsight achieves an average accuracy of 93.2%, surpassing contemporary methods by 5.1 percentage points. The model successfully distinguishes 50 distinct immune cell subtypes with high accuracy, demonstrating proficiency for identifying rare and closely related cell subtypes. Additionally, SHAP-based interpretability quantifies individual gene contributions to reveal the biological basis of classification decisions. These qualities make scHDeepInsight a robust tool for high-resolution cell subtype characterization, well-suited for detailed profiling in immunological studies and extensible to nonimmune cell types.

## Introduction

Comprehensive understanding of complex biological systems and their abnormal states, such as cancer and chronic diseases, requires accurate identification of cell types [2], particularly of immune cells. The advent of single-cell RNA sequencing (scRNA-seq) technology has revolutionized our ability to analyze gene expression profiles at an unprecedented resolution, enabling detailed characterization of cellular heterogeneity at the individual cell level. Widely used methods for cell type annotation, such as SingleR [1], Azimuth [2], and scmap [3], are reference-based, assigning cell type labels by comparing query cells to known reference profiles using similarity metrics. These comparisons are generally processed in a uniform manner, without explicit consideration of the hierarchical relationships among cell types. While such existing annotation tools achieve high accuracy in broad cell type identification, incorporating predefined immune cell hierarchies has the potential to further enhance their effectiveness for detailed subtype classification. By incorporating these hierarchies, it becomes possible to distinguish closely related subtypes, cells which often share high transcriptional similarity yet differ significantly in function and biological roles. Such refinement enhances the biological relevance and interpretability of downstream analyses. We posit that explicitly modeling the known biological hierarchy of immune cell populations in the annotation process is needed. Incorporating hierarchical modeling naturally captures functional relationships and widely accepted immune lineage structures, which are derived from curated ontological annotations and partially aligned with known differentiation patterns, thereby enhancing the biological accuracy and context-specific interpretation of annotation results.

To realize these important improvements, we introduce scHDeepInsight, an enhanced deep learning framework built upon the foundation of scDeepInsight [4] by explicitly employing a hierarchical annotation architecture. The core innovation of scHDeepInsight lies in the integration of three key components: (i) the transformation of high-dimensional gene expression data into spatially-organized two-dimensional (2D) images [5], making them suitable for convolutional neural network (CNN) [6] processing; (ii) the implementation of a multi level classification architecture that preserves the biological hierarchies of base-types and subtypes of cells; and (iii) the incorporation of an adaptive hierarchical focal loss (AHFL) function that automatically balances training priorities by adjusting the weights of base-type and subtype focal losses according to their relative performance. This approach enables researchers to precisely dissect the complexities of the immune system at a higher resolution. Furthermore, the multilevel classification structure enables stratified feature importance quantification through SHAP (SHapley Additive exPlanations) [7] analysis at both base-type and subtype cell levels, thereby providing biological insights into the distinct gene expression signatures that differentiate closely related immune cell populations.

While scHDeepInsight is primarily designed and trained for immune cell classification, its hierarchical architecture is inherently generalizable. The model’s structure allows extension to other cellular contexts, including stromal and epithelial cells, which are particularly relevant in complex tissue environments. This adaptability broadens the utility beyond immunology, making it suitable for integrated annotation tasks in diverse biomedical applications.

## Materials and methods

### Data collection

The scHDeepInsight framework was developed and validated using scRNA-seq data from 10 published studies [2, 8–16], covering a wide range of tissues (e.g. blood, lung, intestine), as detailed in Supplementary Table S1.

**Figure 1:**
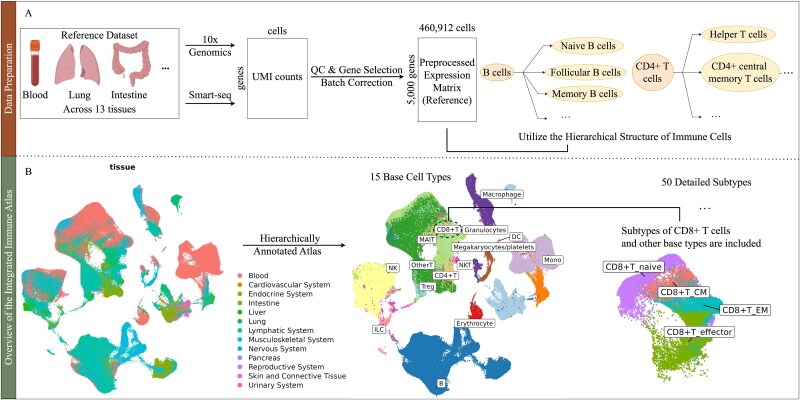
(a) Schematic of data integration, from study selection through quality control (QC), normalization, and batch correction. (b) Low-dimensional visualization of the final reference atlas comprising 15 base cell types and over 50 subtypes.

In total, the reference dataset includes over 460,000 cells from healthy donors, providing a robust baseline for investigating immune cell heterogeneity. After rigorous preprocessing and STACAS-based [17] batch correction (as described in the Supplementary_Note), we constructed an integrated reference atlas centered on the top 5000 highly variable genes extracted via Scanpy [18] from the reference datasets, encompassing 15 base immune cell types and more than 50 subtypes. The data integration pipeline follows a systematic workflow from initial study selection through quality control, normalization, and batch correction (Fig. 1a, Supplementary_Note). The resulting low-dimensional embedding of the reference atlas shows distinct clusters corresponding to different immune cell types and subtypes (Fig. 1b). This hierarchical organization preserves immune cell lineage relationships, ensuring clear separation between major lineages while maintaining biological continuity among related subtypes.

The query (test) datasets utilized in this study for the evaluation were sourced from various public databases, each covering different tissues and cell types. The datasets include peripheral blood mononuclear cells (PBMCs), lung and liver tissue samples, and bone marrow cells (Table 1). They were generated using a range of scRNA-seq protocols, including multiple versions of 10× Genomics [19] (3′ v2, v3) and MARS-seq [20].

**Table 1:** Summary of datasets used for benchmarking scHDeepInsight and the other tools. Gene coverage percentage indicates the proportion of genes in each query dataset that overlaps with the 5000 highly variable genes used in the model training.

Dataset	Tissue	Protocol	Cell numbers	Gene coverage	Cell types
Arunachalam *et al*., 2020	Blood	10× Multiome 3′ v2, v3	25,954	99.8%	22
Lee *et al*., 2020	Blood	10× Multiome 3′ v3	16,298	99.5%	23
Schulte-Schrepping *et al*., 2020	Blood	10× Multiome 3′ v2, v3	45,787	99.6%	24
Adams *et al*., 2020	Lung	10× Multiome 3′ v2	10,934	96.4%	21
Travaglini *et al*., 2020	Lung	10× Multiome 3′ v3	35,699	94.6%	24
MacParland *et al*., 2018	Liver	10× Multiome 3′ v2	4,436	91.2%	18
Ledergor *et al*., 2018	Bone Marrow	MARS-seq	6,367	96.3%	6

### Overview of scHDeepInsight

scHDeepInsight is a computational framework for single-cell data that enables hierarchical immune cell annotation, rare cell-population detection, and biological interpretation through SHAP analysis. The framework applies a structured multilevel classification process that reflects known immune cell lineage relationships and supports fine-grained subtype resolution.

**Figure 2:**
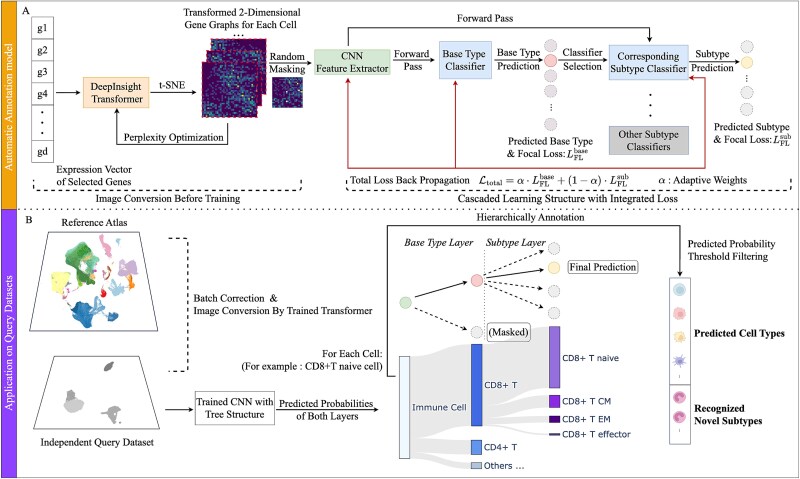
Overview of the scHDeepInsight framework. (a) each cell's gene expression vector is transformed into a 2D image via DeepInsight [5] (t-distributed stochastic neighbor embedding (t-SNE) [21] with perplexity optimization), optionally masked for data augmentation, then fed into an EfficientNet-B5 [22] CNN trained with a multilevel loss to classify cells hierarchically. (b) for query data, after batch correction and image conversion, the trained CNN outputs base-type and subtype probabilities. Masking excludes irrelevant subtypes, enabling hierarchical annotation and uncovering potentially new immune populations.

As illustrated in Fig. 2, the workflow consists of two main stages: (i) Training phase—gene expression vectors are transformed into 2D images via DeepInsight followed by random masking, and then processed by a CNN feature extractor and the hierarchical classification architecture with integrated loss function for model training. (ii) Application phase—scHDeepInsight performs batch effect correction on query datasets by transforming them using the analogous image conversion procedure followed by the hierarchically-trained CNN to predict both primary cell types and their subtypes.

During the training phase, scHDeepInsight transforms preprocessed gene expression profiles into two-dimensional gene expression images constructed from a reference atlas. These images are then used to train a CNN based on the EfficientNet-B5 architecture. Unlike the original scDeepInsight, which employs a flat classification approach, scHDeepInsight integrates a multilevel loss function that preserves and reinforces the hierarchical relationships between cell types during classification. The architecture enables a two-stage classification process: first identifying the primary immune cell types, then further refining subtype classification within each lineage.

In the application (test) phase, scHDeepInsight is applied to independent query datasets. The batch-corrected datasets are then fed into the pretrained CNN, which outputs predicted probabilities for both primary cell types and subtypes. For rare cell identification, scHDeepInsight employs a probability-based detection mechanism that analyzes the discrepancy patterns between base-type and subtype prediction confidences.

### Conversion of tabular data into images

To transform high-dimensional scRNA-seq data into CNN-compatible 2D image representations, pyDeepInsight tool (https://github.com/alok-ai-lab/pyDeepInsight), based on the DeepInsight framework [23], is employed. In this approach, each gene is mapped to a specific pixel location, and pixel intensity reflects the corresponding gene expression level. Manifold techniques, such as t-SNE and Uniform Manifold Approximation and Projection (UMAP) [24], are applied with optimized perplexity settings to project the data into a 2D space, positioning genes with similar expression patterns in close proximity. These coordinates are then converted into pixel positions, creating images in which the intensities represent gene expression patterns. Considering that query datasets may lack certain genes present in the reference atlas used for training, random masking is introduced to the generated images, thereby injecting controlled noise to enhance robustness against missing gene features in query data. The resulting 2D representations (224 × 224 × 3) are then utilized by an EfficientNet-B5 CNN for feature extraction and cell type classification. Furthermore, to ensure an optimal gene-to-pixel assignment and eliminate potential collisions where multiple genes would be mapped to the same pixel location, a Linear Sum Assignment (LSA) algorithm was applied [25]. This image-based approach leverages the CNN’s capacity for feature learning while preserving the spatial structure among genes, thereby capturing the underlying gene–gene interaction patterns.

### Adaptive hierarchical focal loss

For effective hierarchical classification, the model must address class imbalance, which is commonly observed in single-cell RNA-seq datasets where rare immune cell subtypes can be underrepresented by orders of magnitude compared to abundant populations, while maintaining predictive accuracy across different granularity levels. To achieve this, scHDeepInsight employs an AHFL that extends the focal loss framework [26] to optimize classification at multiple levels of the hierarchy. For both base-type and subtype classification levels, the focal loss $FL(p)$ is defined as:


(1) \begin{equation*} FL(p)=-{\sum}_{i=1}^K{y}_i{\left(1-{p}_i\right)}^{\gamma}\mathit{\log}\left({p}_i\right) \end{equation*}

In Equation 1, $K$ represents the total number of possible classes at the respective classification level (15 base types or their corresponding subtypes), $p$ is the predicted probability vector, where ${p}_i\in \left[0,1\right]$ corresponds to the predicted probability for the $i$th class, ${y}_i\in \left\{0,1\right\}$ denotes the ground truth binary label for class $i$ (1 if the sample belongs to class $i$, 0 otherwise) and $\gamma$ is the focusing parameter that modulates the influence of well-classified versus hard-to-classify examples. We set $\gamma =2.0$ in all experiments, adopting the optimal value reported in the original focal loss paper [26] through empirical validation for addressing class imbalance in object detection tasks.

The AHFL adapts the focal loss to operate simultaneously at two hierarchical levels: base-type classification and subtype classification. The total loss function combines these two levels as:


(2) \begin{equation*} {L}_{total}=\alpha \cdotp{FL}^{base}+\left(1-\alpha \right)\cdotp{FL}^{sub} \end{equation*}

The variables ${FL}^{base}$and ${FL}^{sub}$ represent the focal losses computed for base-type and subtype classification respectively, calculated using Equation 1 with corresponding labels and predictions. The weighting parameter $\alpha \in \left[0,1\right]$ balances their relative contributions. Unlike static weighting schemes, $\alpha$ is dynamically updated during training based on the relative performance of the two classification levels. The adaptive weighting mechanism is formalized as:


(3) \begin{equation*} {\alpha}_{t+1}=\beta \cdotp{\alpha}_t+\left(1-\beta \right)\cdotp \frac{FL^{base}}{FL^{base}+{FL}^{sub}} \end{equation*}

Here, ${\alpha}_t$ is the adaptive weight at training step $t$, and, ${\alpha}_{t+1}$is the updated weight for the next iteration. The momentum parameter $\beta \in \left[0,1\right]$ controls the smoothness of weight adaptation and is set to 0.9, following standard practices in adaptive weighting schemes [27], to provide stable exponential smoothing that balances historical of ${\alpha}_t$ values with responsiveness to current loss ratios. The ratio of ${FL}^{base}/\left({FL}^{base}+{FL}^{sub}\right)$serves as the adaptation signal that drives this dynamic adjustment. The adaptive weighting mechanism dynamically directs the model's focus towards the most challenging hierarchical level during training. When base-type classification becomes more accurate (indicated by lower ${FL}^{base}$), the algorithm shifts focus in the next iteration toward improving subtype classification by decreasing ${\alpha}_{t+1}$. Conversely, when subtype classification improves substantially, the model allocates more weight to refining base-type predictions. Through joint backpropagation, the framework simultaneously optimizes both classification levels, improving overall accuracy while maintaining consistency with known immune cell hierarchies.

### Hierarchical prediction model

scHDeepInsight integrates immune lineage structure directly into its classification architecture and training process. This design allows the model to make structured predictions that reflect known biological organization (the complete hierarchical organization of immune cell types and their relationships is visualized in Supplementary Fig. S1).

**Figure 3:**
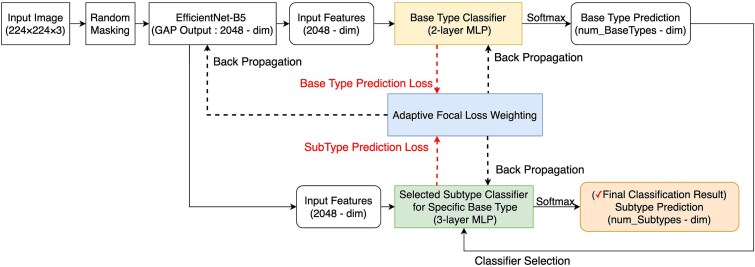
Hierarchical classification model for single-cell type identification. The model uses EfficientNet-B5 to extract features from transformed gene expression images for sequential classification of cell types (base-types) and subtypes. The adaptive focal loss weighting mechanism addresses class imbalance while balancing optimization between base-type and subtype levels.

After image conversion and feature extraction with CNN, these extracted features are processed through a hierarchical classification pipeline (Fig. 3). The base-type classifier first identifies the broad immune cell category, followed by activation of the corresponding subtype classifier for refined identification within that lineage (detailed architecture illustrated in Supplementary Fig. S2).

During the training phase, a multilevel focal loss function optimizes predictions at both base-type and subtype levels simultaneously, with error signals propagated back through the network to capture hierarchical dependencies between cell types.

During prediction, probability masking sets the probabilities of subtypes outside the predicted primary category to zero, ensuring that downstream classifiers operate only within biologically relevant subtype spaces and reducing misclassification across unrelated lineages. In cases where a base-type has no defined subtypes, it is treated as a terminal leaf in the classification tree. The model bypasses the subtype classifier for such nodes, and the base-type prediction itself serves as the final output. Terminal node treatment preserves hierarchical consistency while accommodating the limited subtype resolution available in current immune reference annotations.

## Results

### Benchmarking with state-of-the-art methods

To evaluate the performance of scHDeepInsight (Methods), a series of benchmarking experiments were conducted using seven independent query datasets. These datasets were selected to represent a diverse array of tissues, cell types, and disease conditions, providing a testbed for assessing the accuracy, precision, and robustness of scHDeepInsight compared to other state-of-the-art (SOTA) cell annotation methods, including SingleR [1], Azimuth [2], scDeepInsight [4], CellTypist [8], Garnett [28], scType [29], and GPTCellType [30]. The evaluation metrics and technical summaries of these benchmarked methods are provided in the Supplementary_Note.

#### Benchmarking evaluation across multiple metrics

**Figure 4:**
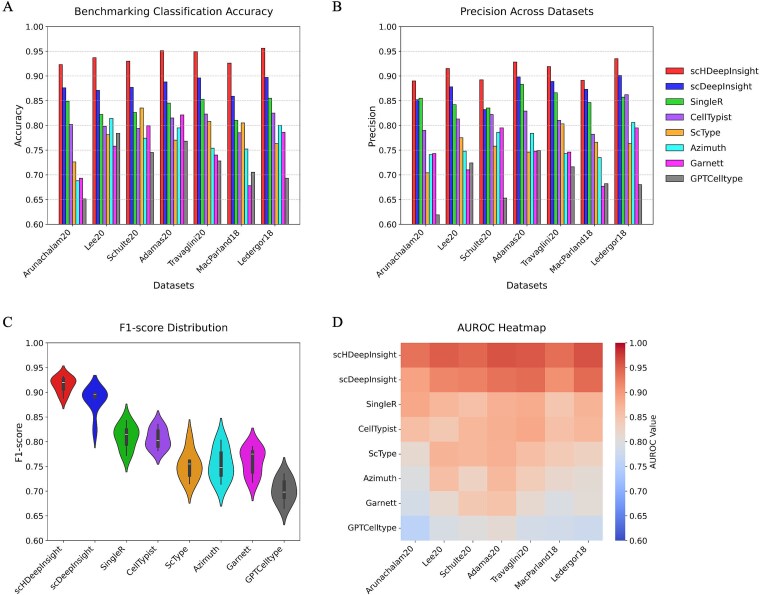
Benchmarking results. (a) Accuracies across seven datasets for all classification methods (scHDeepInsight in red). (b) Precisions for the same methods. (c) F1-score distributions as violin plots revealing median performance and variability across methods. (d) AUPRC heatmap displaying classification strength with color intensity corresponding to performance values.

Comprehensive benchmarking was performed at both base-type and subtype classification levels. At the subtype level, scHDeepInsight demonstrated consistent robust performance, achieving an average accuracy of 93.2% and precision of 91.1% across diverse datasets (Fig. 4A and B; the detailed training and validation accuracy trends are provided in Supplementary Fig. S3). The method achieved an F1-score of 90.5%, reflecting a balance between precision and recall, alongside an area under the precision-recall curve (AUPRC) of 89.7%, a metric particularly suitable for evaluating classification performance on datasets with imbalanced class distributions (Fig. 4C and D). Comparative analysis with scDeepInsight (the next highest performing method) revealed improvements of 5.1% in accuracy, 3.3% in precision, 3.1% in F1-score, and 3.6% in AUPRC (detailed comparative results for all methods are shown in Supplementary Table S2). These improvements across all evaluation metrics indicate scHDeepInsight's enhanced classification capability across the diverse cell types and datasets tested. Technical strategies for rare subtype handling and overfitting prevention are detailed in Supplementary_Note.

At the base-type level, scHDeepInsight achieved 96.8% accuracy, 95.9% precision, 97.0% F1-score, and 93.5% AUPRC (Supplementary Fig. S4). Compared to the second-best method scDeepInsight, this represents improvements of 2.5, 1.6, 2.4, and 2.7 percentage points in accuracy, precision, F1-score, and AUPRC, respectively. The improvements were greater at the subtype level compared to the base-type level across all metrics, indicating that the hierarchical framework provides enhanced benefits for fine-grained classification tasks.

#### Fine-grained immune cell classification

**Figure 5:**
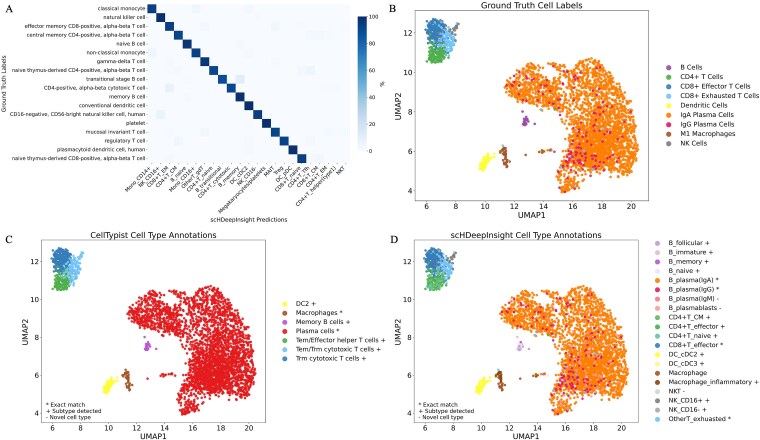
Classification results demonstrating immune cell subtype identification capabilities. (a) the confusion matrix of scHDeepInsight predictions on the Lee dataset. (b) UMAP visualization of the labial gland dataset with original expert annotations, including detailed plasma cell subtypes. (c) Annotation results with CellTypist on the labial gland dataset. (d) scHDeepInsight classification results on the labial gland dataset, with symbols indicating correspondence to the original annotations.

Owing to the hierarchical classification design and comprehensive reference atlas covering 50 immune cell subtypes, scHDeepInsight demonstrates precise distinction of closely related subtypes. For instance, in the PBMC query dataset (Lee) [31], scHDeepInsight successfully distinguished between closely related subtypes, achieving high classification accuracy with minimal misclassification errors (Fig. 5A). In the labial gland dataset Pranzatelli [32], scHDeepInsight successfully recovered the distinct immunoglobulin-based subtypes (IgA+ and IgG+) of plasma cells originally labeled by experts (Fig. 5B and D), whereas, other supervised annotation methods failed to maintain this resolution, instead grouping all plasma cells into a single broad category (Fig. 5C). By delineating these subtle transcriptional differences, scHDeepInsight highlights the distinct advantage provided by the hierarchical classification framework in accurately resolving closely related immune cell populations.

### Recognition of rare cell types

The hierarchical classification framework of scHDeepInsight enables novel cell population detection through analysis of prediction probability patterns: the model assigns probability scores at both base and subtype levels and creates a quantitative signature that reflects cellular identity with greater nuance than conventional binary classification approaches (Methods).

**Figure 6:**
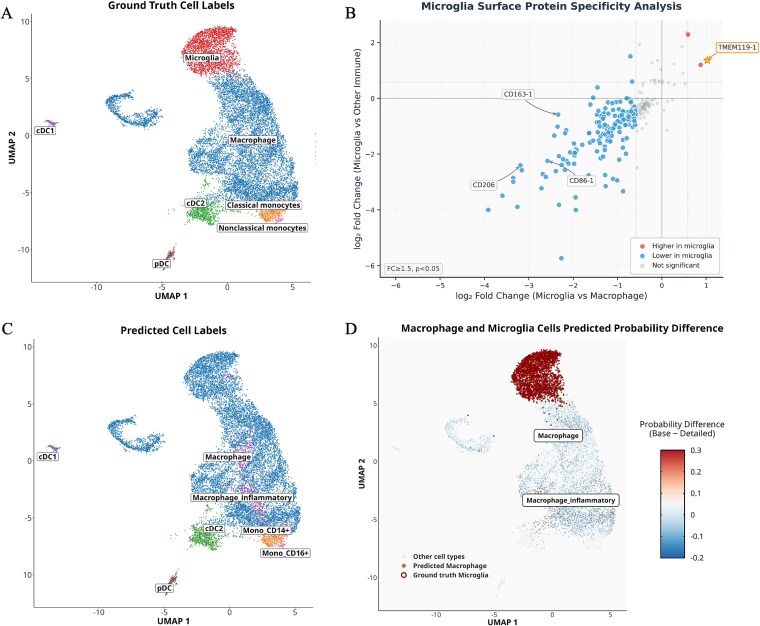
Identification of novel cell populations through hierarchical probability analysis validated with CITE-seq data. (a) UMAP visualization with ground truth cell annotations displaying microglia and macrophage populations. (b) Surface protein expression analysis demonstrating differential expression of microglia-specific (TMEM119) and macrophage-specific (CD163, CD206, CD86) markers. (c) scHDeepInsight classification results for the corresponding cell populations. (d) Hierarchical probability difference analysis (base-type minus subtype probabilities) highlighting potential novel cell states.

Validation using CITE-seq [33] data from a brain immune cell atlas [34] demonstrates the model's ability to detect novel cell populations through hierarchical probability patterns. Ground truth annotations reveal distinct microglia and macrophage populations (Fig. 6A), which are supported by differential surface protein expression: microglia exhibit higher TMEM119 expression while macrophages show elevated CD163, CD206, and CD86 (Fig. 6B). Our model predictions successfully identified these populations as macrophage lineage cells (Fig. 6C), with hierarchical probability difference analysis revealing the key signature of novel cell states: regions with high base-type probabilities for this lineage but low subtype-level confidence (Fig. 6D). This pattern, particularly evident in microglia-enriched regions, indicates cells that belong to the broader myeloid lineage but represent subtypes beyond the training atlas scope.

These findings illustrate that scHDeepInsight's probabilistic output supports a graded representation of cellular identity, capturing both canonical immune subtypes and cell populations that diverge from transcriptional profiles represented in the reference atlas.

### Analysis of SHAP-based feature importance for immune cell classification

**Figure 7:**
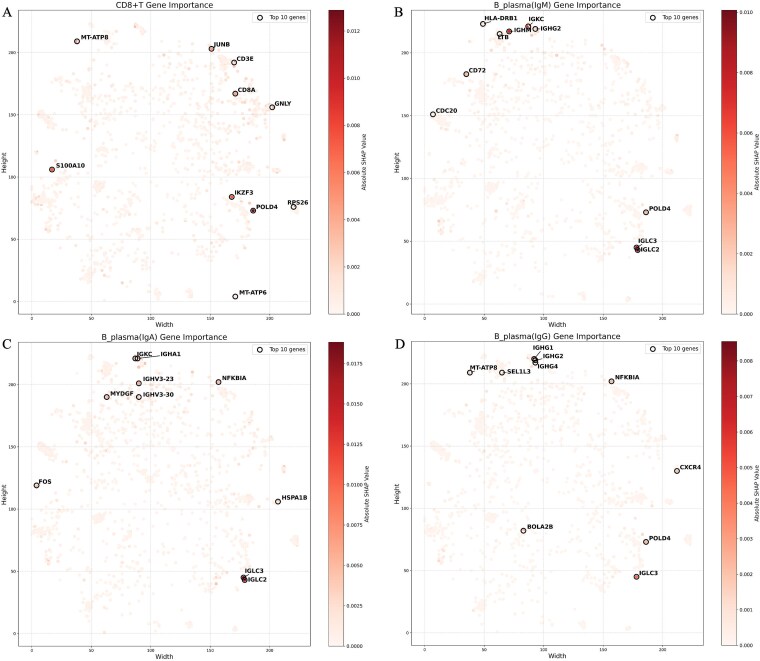
SHAP-based gene importance analysis for immune cell classification. (a) CD8^+^ T-cell gene importance, highlighting CD8A, CD3E, and effector molecules like GNLY. (b) IgM-expressing plasma cell gene importance, with high values for IGHM. (c) IgA-expressing plasma cell gene importance, showing elevated SHAP values for IGHA1 and associated light chain genes. (d) IgG-expressing plasma cell gene importance, featuring IGHG1, IGHG2.

To identify discriminative gene features critical for accurate cell type classification, scHDeepInsight employs SHAP analysis separately for each immune cell type and subtype, whose absolute value quantitatively measures the contribution of each gene to the model's classification decisions. The resulting values represent the average feature importance across cells within each class, thereby enabling identification of both lineage-defining and subtype-specific gene signatures. As shown in Fig. 7, the model identifies distinct gene importance patterns across different immune cell populations, with both expected canonical markers and subtype-specific genes. For instance CD8+ T-cells (Fig. 7A), canonical T-cell markers such as CD8A and CD3E demonstrate high positive SHAP values, reaffirming their expected roles in defining cytotoxic T-cell identity. Similarly, the cytotoxic effector molecule GNLY and transcription factors associated with T-cell activation contribute positively to classification decisions. Notably, SHAP analysis captures genes with reduced expression that characterize cell subtypes. For example, CD72, which is typically downregulated during plasma cell differentiation [35], is identified as an important discriminative feature in IgM-expressing plasma cells (Fig. 7B), consistent with known biological phenomena. The model also successfully identifies common gene features of plasma cells across subtypes, such as immunoglobulin light chain genes (IGLC2, IGLC3), while capturing the isotype-specific differences that biologically distinguish these populations: IGHA1 for IgA-expressing plasma cells (Fig. 7C), IGHM for IgM-expressing plasma cells (Fig. 7B), and IGHG1/IGHG2 for IgG-expressing plasma cells (Fig. 7D). These findings validate that the detailed subtype classifier within the hierarchical classification framework in scHDeepInsight can capture both lineage-shared and subtype-specific genes, further supporting its ability to discriminate between closely related cellular states from an immunological perspective.

## Discussion

Accurate identification of broad cell types as well as subtypes of immune cells is a prerequisite for understanding their diverse roles in complex biological systems and diseases, such as cancer and chronic conditions. scHDeepInsight advances immune cell annotation in scRNA-seq data by implementing a hierarchical classification framework that reflects the biological organization of immune lineages. In contrast to conventional annotation strategies that treat all cell types as independent categories, scHDeepInsight leverages shared representations across hierarchical levels, thereby improving the resolution of both major lineages and transcriptionally similar subtypes.

Beyond improvements in predictive performance, scHDeepInsight incorporates several innovations designed to enhance biological interpretability. The AHFL dynamically balances optimization across classification levels, increasing sensitivity to both coarse-grained and fine-grained cell distinctions. By transforming gene expression profiles into spatially organized images, a CNN can learn complex expression patterns from inherently nonspatial data. Additionally, the model produces structured probability distributions across the hierarchy, providing a continuous and interpretable representation of cellular identity. This probabilistic framework enables identification of cells with ambiguous subtype assignments, which may represent novel cell populations, or context-specific immune phenotypes. Such cases are exemplified in the glioblastoma dataset, where cells demonstrate high confidence in base-type classification but exhibit uncertainty in subtype assignment. Model interpretability is further strengthened by SHAP-based feature analysis, which reveals gene expression patterns associated with both lineage specification and functional divergence. By identifying canonical markers alongside context-specific regulatory features, the model not only enhances confidence in subtype predictions but also provides insights into the gene expression patterns underlying immune heterogeneity. However, interpretation of SHAP-based feature importance should take into account the inherent gene dropout and sparsity in scRNA-seq data, particularly when evaluating negative feature contributions.

While scHDeepInsight presents significant advances, certain challenges remain. Classification performance is fundamentally linked to the resolution and completeness of the reference atlas, which may limit annotation in cases of sparse or incomplete reference data. Computational benchmarking on a 10,000-cell scRNA-seq dataset demonstrates that scHDeepInsight achieves 72 s runtime and 6.2 GB peak memory usage, which is comparable to scDeepInsight and more efficient than several baseline methods including SingleR and CellTypist (Supplementary Fig. S5). Despite its additional computational cost compared to lightweight methods such as scType, the substantial performance improvements justify the overhead for applications requiring high-resolution immune cell profiling.

In future work, further development of scHDeepInsight will focus on extending its generalizability and biological scope. While the current model is trained on immune cell types, the hierarchical classification architecture is generalizable to other cellular contexts. To demonstrate this potential, we trained a separate model on a breast cancer dataset [36] (GSE176078, *n* = 100,064 cells) containing both immune and nonimmune cell types. Cross-validation results show that the framework achieves robust performance across diverse cell populations including epithelial, stromal, and immune cells, with an overall accuracy of 90.7% (Supplementary Fig. S6), indicating the architectural adaptability of scHDeepInsight to diverse cellular systems beyond the immune compartment. Additionally, integration of multi-omics modalities, such as CITE-seq and spatial transcriptomics, may improve subtype resolution by incorporating protein-level and spatial context information. These complementary data types would also enhance the reliability and interpretability of SHAP-based feature importance analysis by providing orthogonal validation of gene expression patterns identified as discriminative features. Also, self-supervised learning approaches may facilitate feature extraction from unlabeled data, enabling discovery of novel immune states without prior annotation. Incorporating pseudotime inference would allow dynamic modeling of differentiation trajectories, extending the framework beyond static classification. Finally, transfer learning strategies offer a promising path toward improving adaptability across tissues or species with minimal retraining, broadening the applicability of scHDeepInsight to diverse biological contexts. As a further extension, incorporating nonimmune cell populations into the hierarchical framework would enable unified modeling of diverse cellular systems beyond the immune compartment.

## Conclusion

scHDeepInsight provides a deep learning framework for hierarchical immune cell classification using scRNA-seq data, incorporating CNN architectures and immune lineage structures to enhance accuracy and resolution of cell type annotation. Through the integrated use of the multilevel loss function and adaptive weighting approach, it achieves accurate identification of both common immune lineages and closely related subtypes while maintaining their hierarchical relationships. The integration of batch effect correction ensures consistent performance across datasets under diverse experimental conditions. The comprehensive benchmarking demonstrated that scHDeepInsight outperformed the existing annotation methods across the multiple performance metrics, particularly in distinguishing the closely related immune cell subtypes within complex tissues. In addition, scHDeepInsight revealed novel immune populations through hierarchical analysis on prediction probabilities, highlighting its potential for uncovering biologically relevant cellular diversity beyond canonical annotations. This improved resolution enables precise characterizations of cellular heterogeneities in immunological research.

As the single-cell technologies continue to evolve, the hierarchical classification approach implemented in scHDeepInsight will be increasingly valuable for advancing our understanding of immune cell diversity and functional specialization. The framework's proven adaptability to nonimmune cell types further extends its utility for broad single-cell annotation applications beyond immunological research.

**Key Points**

- scHDeepInsight introduces a deep learning framework that leverages biologically informed hierarchical classification and an adaptive hierarchical focal loss, which is demonstrated to deliver superior performance for cell type detection in single-cell RNA-seq data.
- This architecture addresses the limitations of flat classification, more specifically for the tasks of high-resolution annotation of transcriptionally similar or rare immune subtypes.
- Evaluation on seven independent datasets achieved the highest overall accuracy (93.2%) among seven alternative methods, surpassing the second-best performing method by 5.1 percentage points, and outperforming all others in precision, F1-score, and AUPRC.
- Feature importance analysis using SHAP identified both canonical and subtype-specific gene signatures consistent with established immune cell hierarchies, validating the biological relevance of these predictions and showcasing how this method can improve functional interpretation of immune heterogeneity.
- The complete pipeline is available as a fully functional Python package. The source code and reference datasets are also made available to facilitate reproducibility and reuse.

## Supplementary Material

Supplementary_File_Revised_bbaf523(1)

## Data Availability

All datasets used in this study were obtained from publicly available repositories. The Lee (GSE149689) [31], Pranzatelli (phs002446) [32], Adamas [37] (GSE134692), Travaglini [38] (EGAS00001004344), Arunachalam (GSE155673) [39], Schulte (EGAS00001004571) [40], MacParland (GSE115469) [41] and Ledergor (GSE117156) [42] datasets are also accessible via the CELLxGENE [43] portal: https://cellxgene.cziscience.com/datasets. The integrated reference dataset used for model training is available at Figshare (https://doi.org/10.6084/m9.figshare.28831010).

## Code availability

The source code is publicly available at the GitHub repository: https://github.com/shangruJia/scHDeepInsight. A packaged Python library is also accessible via PyPI (https://pypi.org/project/SCHdeepinsight/) for straightforward installation and use.
