# Supplementary material for: scHDeepInsight: a hierarchical deep learning framework for precise immune cell annotation in single-cell RNA-seq data
Source: Brief Bioinform. 2025 Oct 9;26(5):bbaf523. doi: 10.1093/bib/bbaf523 (PMC12502058; doi:10.1093/bib/bbaf523)
Supplement: Supplementary_File_Revised_bbaf523(1) [file supplementary_file_revised_bbaf523(1).docx]

Supplementary File

**scHDeepInsight: A Hierarchical Deep Learning Framework for Precise Immune Cell Annotation in Single-Cell RNA-seq Data**

Shangru Jia^1^, Artem Lysenko^2,*^ , Keith A Boroevich^3^, Alok Sharma^2,3,4,5,6,*^, Tatsuhiko Tsunoda^1,2,6,*^

^1^ Laboratory for Medical Science Mathematics, Department of Computational Biology and Medical Sciences, Graduate School of Frontier Sciences, The University of Tokyo, Japan.

^2^ Laboratory for Medical Science Mathematics, Department of Biological Sciences, School of Science, The University of Tokyo, Japan.

^3^ RIKEN Center for Integrative Medical Sciences, Japan.

^4^ Institute for Integrated and Intelligent Systems, Griffith University, Australia.

^5^ College of Informatics, Korea University, Seoul, South Korea

^6^ Co-last authors

^*^ Corresponding authors: Artem Lysenko, Laboratory for Medical Science Mathematics, Department of Biological Sciences, School of Science, The University of Tokyo. 7-3-1 Hongo, Bunkyo-ku, Tokyo 113-0033, Japan. E-mail: [alysenko@g.ecc.u-tokyo.ac.jp](mailto:alysenko@g.ecc.u-tokyo.ac.jp); Alok Sharma, Laboratory for Medical Science Mathematics, Department of Biological Sciences, School of Science, The University of Tokyo. 7-3-1 Hongo, Bunkyo-ku, Tokyo 113-0033, Japan. E-mail: [alok.fj@gmail.com](mailto:alok.fj@gmail.com); Tatsuhiko Tsunoda, Laboratory for Medical Science Mathematics, Department of Biological Sciences, School of Science, The University of Tokyo. 7-3-1 Hongo, Bunkyo-ku, Tokyo 113-0033, Japan. E-mail: [tsunoda@bs.s.u-tokyo.ac.jp](mailto:tsunoda@bs.s.u-tokyo.ac.jp)

_
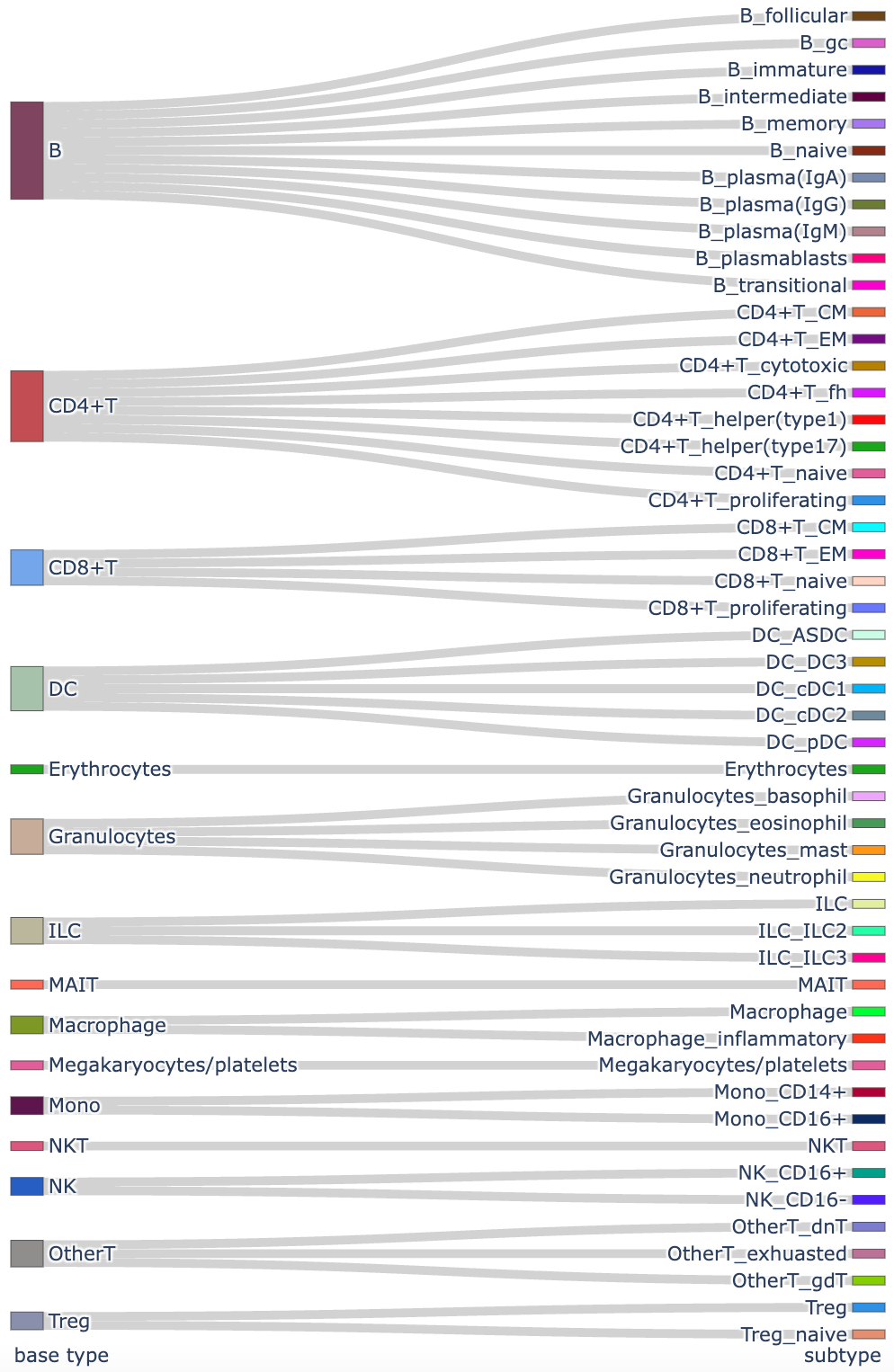
_

**Supplementary_Figure 1: Hierarchical structure of immune cell annotation used in scHDeepInsight.** The Sankey diagram illustrates the reference-based organization of broad immune cell types (base types, left) and their associated subtypes (right) as defined by curated immune ontologies. In cases where no subtypes are available, base types are treated as terminal categories. This structured hierarchy is used to guide the adaptive hierarchical focal loss in scHDeepInsight, improving classification accuracy and biological coherence.


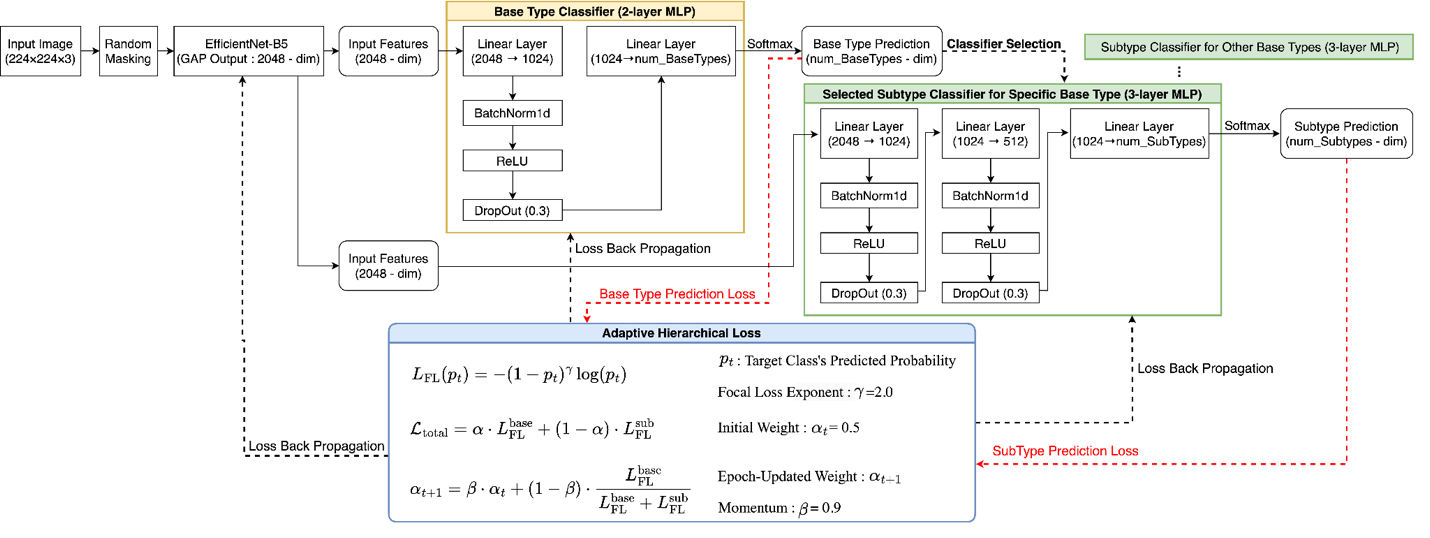


**Supplementary_Figure 2: Detailed architecture of the scHDeepInsight hierarchical classification model.** Input images, generated from gene expression profiles, are processed through an EfficientNet-B5-based feature extractor. Extracted features are hierarchically classified first into base immune cell types via a two-layer MLP classifier, followed by a subtype-specific three-layer MLP classifier. Adaptive hierarchical focal loss (AHFL) dynamically balances the training emphasis between base-type and subtype classification, optimizing hierarchical annotation accuracy. The figure specifies layer dimensions, dropout rates, activation functions, and details of loss calculation.


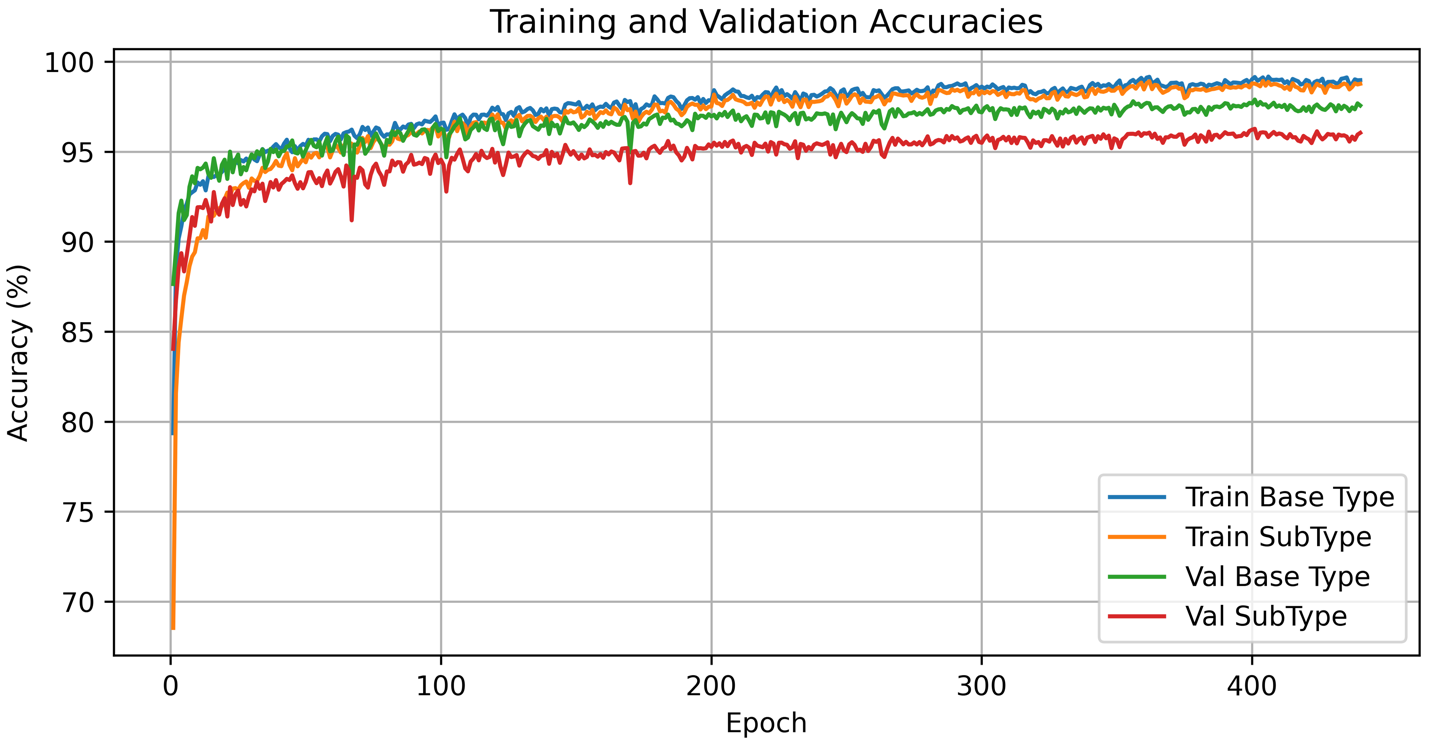


**Supplementary_Figure 3: Training and validation accuracy curves across epochs for scHDeepInsight.** The model's predictive performance for both base immune cell types and finer-grained subtypes is plotted separately, demonstrating stable convergence. Base-type classification (blue and green lines) stabilizes around epochs 100-150, while subtype-level accuracy (orange and red lines) continues to improve gradually, reflecting the challenge of distinguishing closely related immune cell populations. Training extends to ~400 epochs with early stopping (patience=40) to ensure adequate representation of rare cell subtypes. Validation accuracies closely track training accuracies without signs of significant overfitting, confirming robust generalization capabilities of the hierarchical model across epochs, and supporting the chosen training strategy and adaptive hierarchical focal loss implementation.


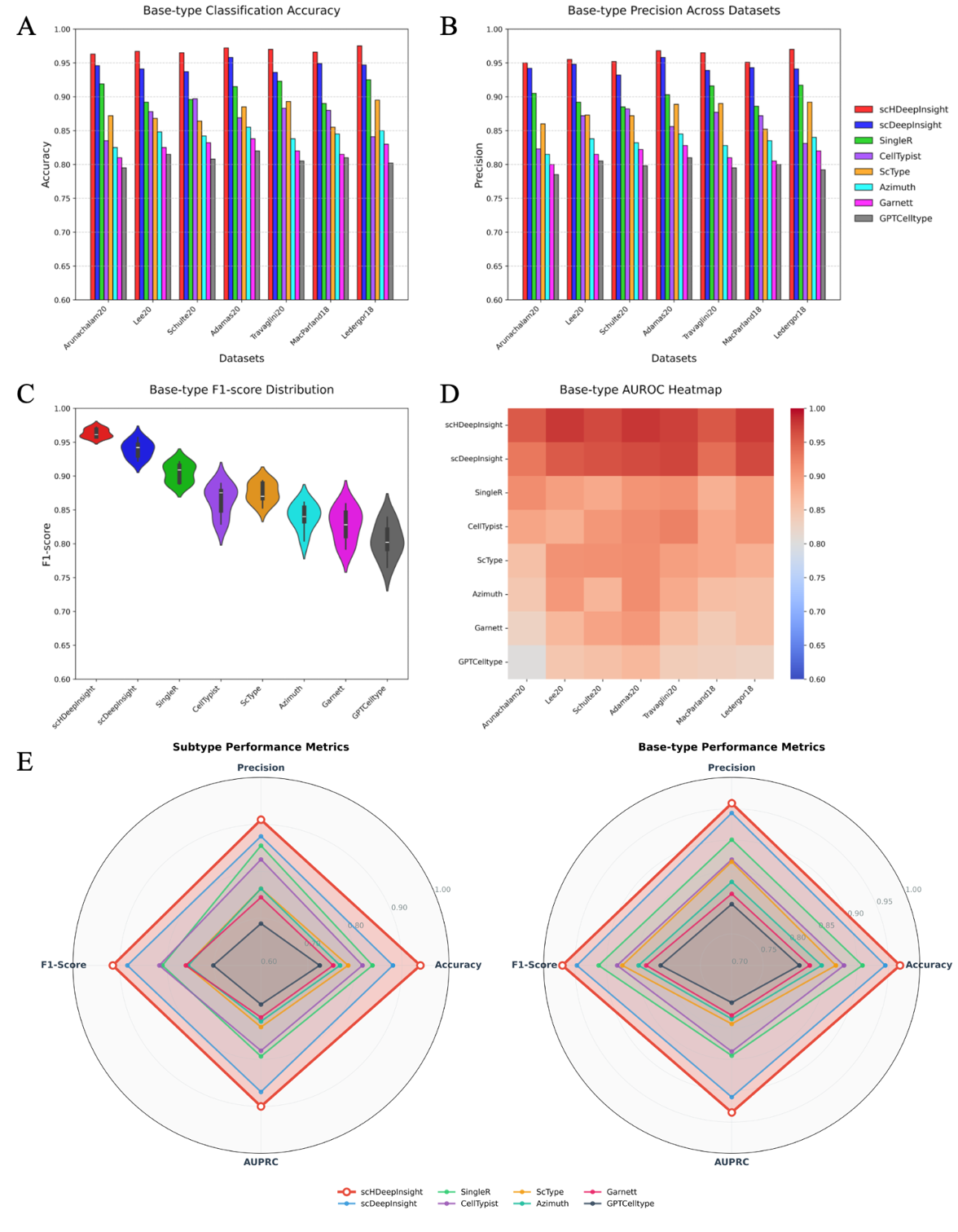


**Supplementary_Figure 4: Base-type classification benchmarking results. (A)** Accuracies and **(B)** precisions across seven datasets for all methods (scHDeepInsight in red). **(C)** F1-score violin plots and **(D)** AUPRC heatmap. **(E)** Radar plots comparing subtype (left) versus base-type (right) performance across all metrics, showing scHDeepInsight's superior performance with greater improvements at the subtype level.

**
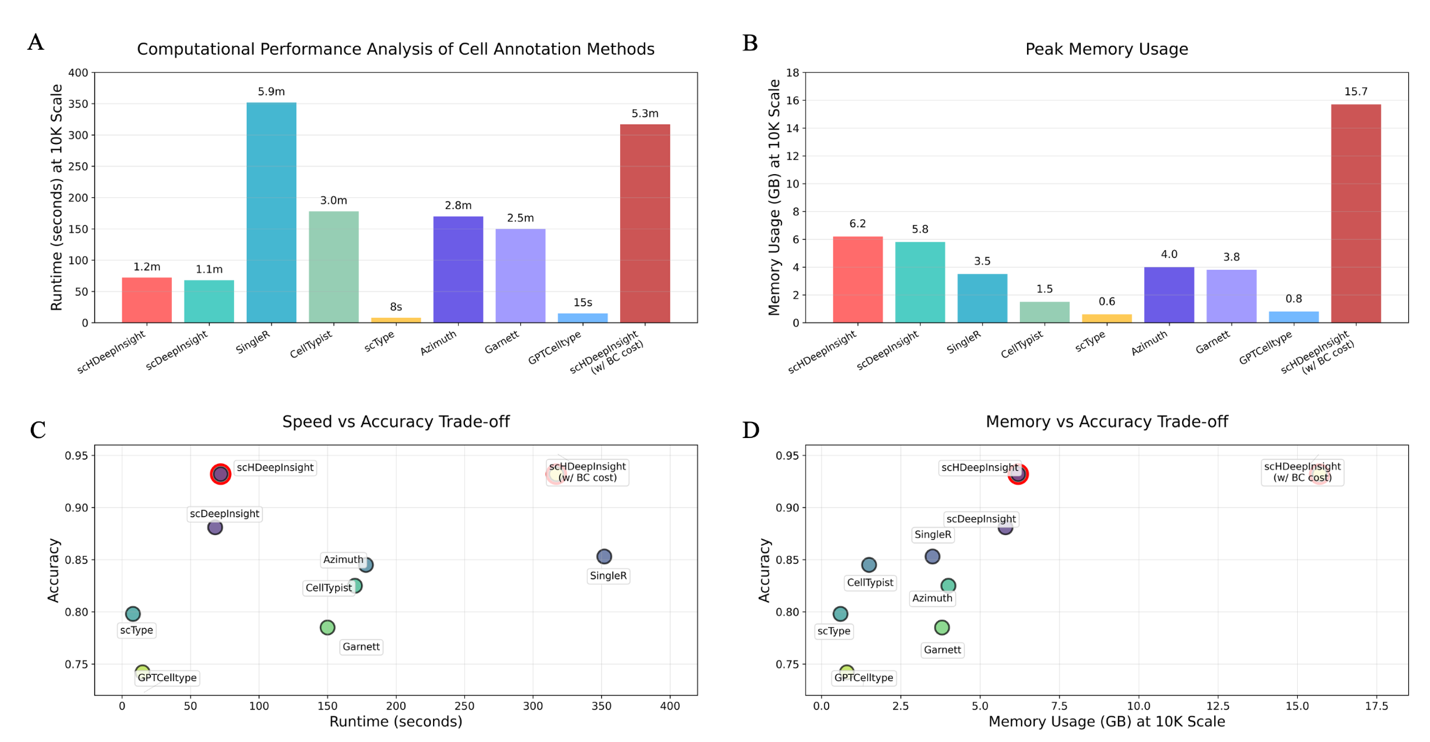
**

**Supplementary_Figure 5: Computational performance analysis of cell annotation methods on a 10,000-cell scRNA-seq dataset (benchmarked on 4×A100 GPUs).** **(A)** Runtime comparison across methods, showing processing time in seconds. **(B)** Peak memory usage in GB. **(C)** Speed-accuracy trade-off analysis, plotting runtime against classification accuracy. **(D)** Memory-accuracy trade-off, showing memory usage versus accuracy. Red circles highlight scHDeepInsight's position, demonstrating competitive computational efficiency while achieving superior accuracy. Note: scHDeepInsight (w/ BC cost) includes batch correction overhead.


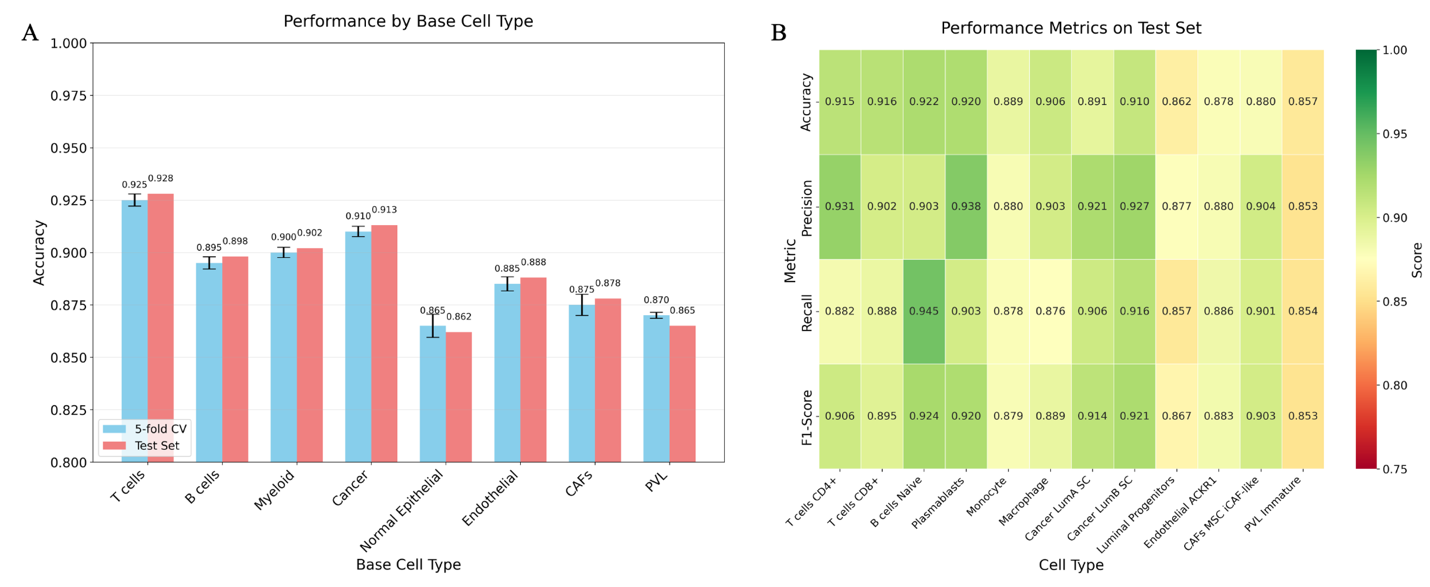
**Supplementary_Figure 6: Validation of scHDeepInsight on breast cancer tissue with mixed immune and non-immune cell populations. (A)** Classification accuracy for eight major cell types in a breast cancer dataset (GSE176078, n=100,064 cells) using 5-fold cross-validation with 75:10:15 train/validation/test split. Error bars indicate standard deviation across CV folds. **(B)** Heatmap of performance metrics (accuracy, precision, recall, F1-score) on the test set for all cell types. The model achieves robust performance across diverse cell populations including immune cells (T cells, B cells, Myeloid), epithelial cells (Cancer, Normal Epithelial), and stromal cells (CAFs, Endothelial, PVL) with overall test accuracy of 90.7%, demonstrating generalizability beyond immune-only datasets.

**
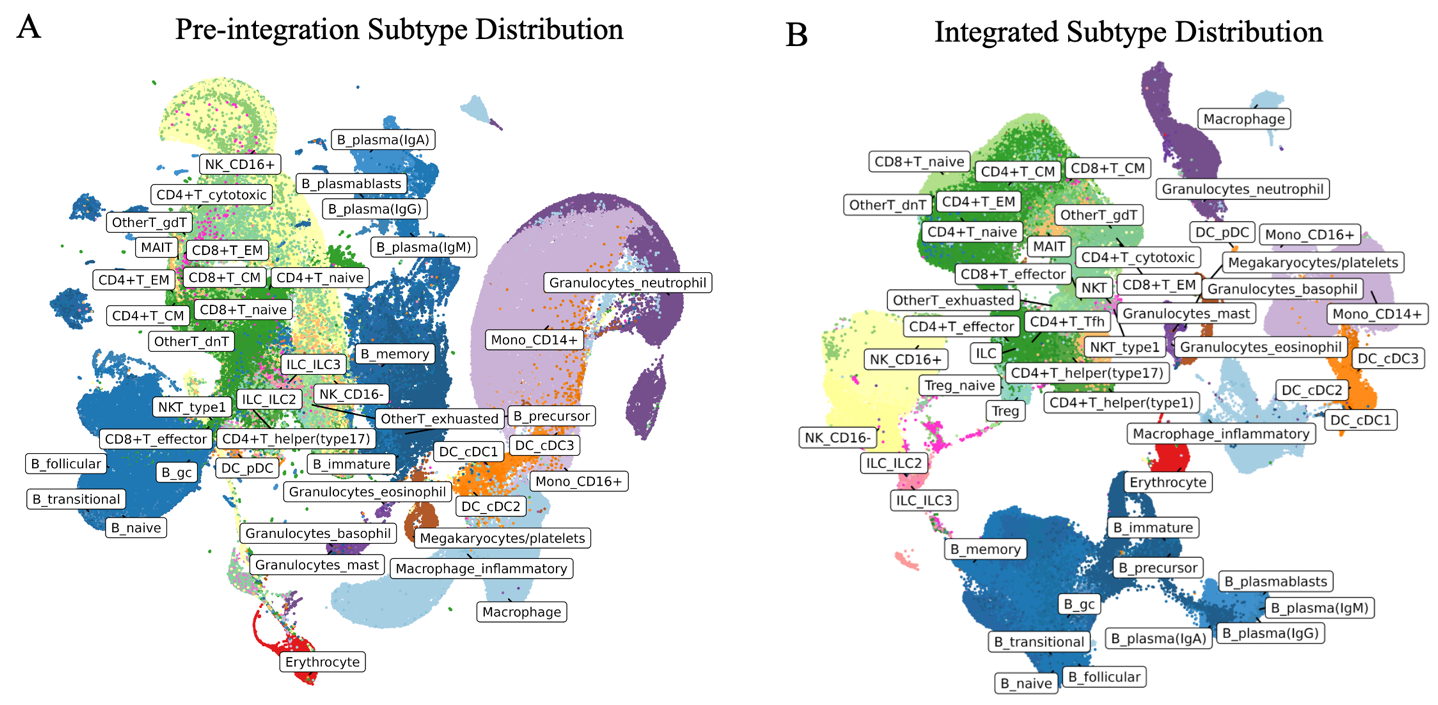
**

**Supplementary_Figure 7:** **Distribution of immune cell types in reference datasets before and after batch effect correction.** **(A)** Pre-integration UMAP of immune subtypes, showing batch-driven dispersion. **(B)** Post-integration UMAP of immune subtypes, demonstrating improved clustering. A subset of the reference dataset was used to project query cells into the integrated reference space, ensuring biologically consistent alignment.


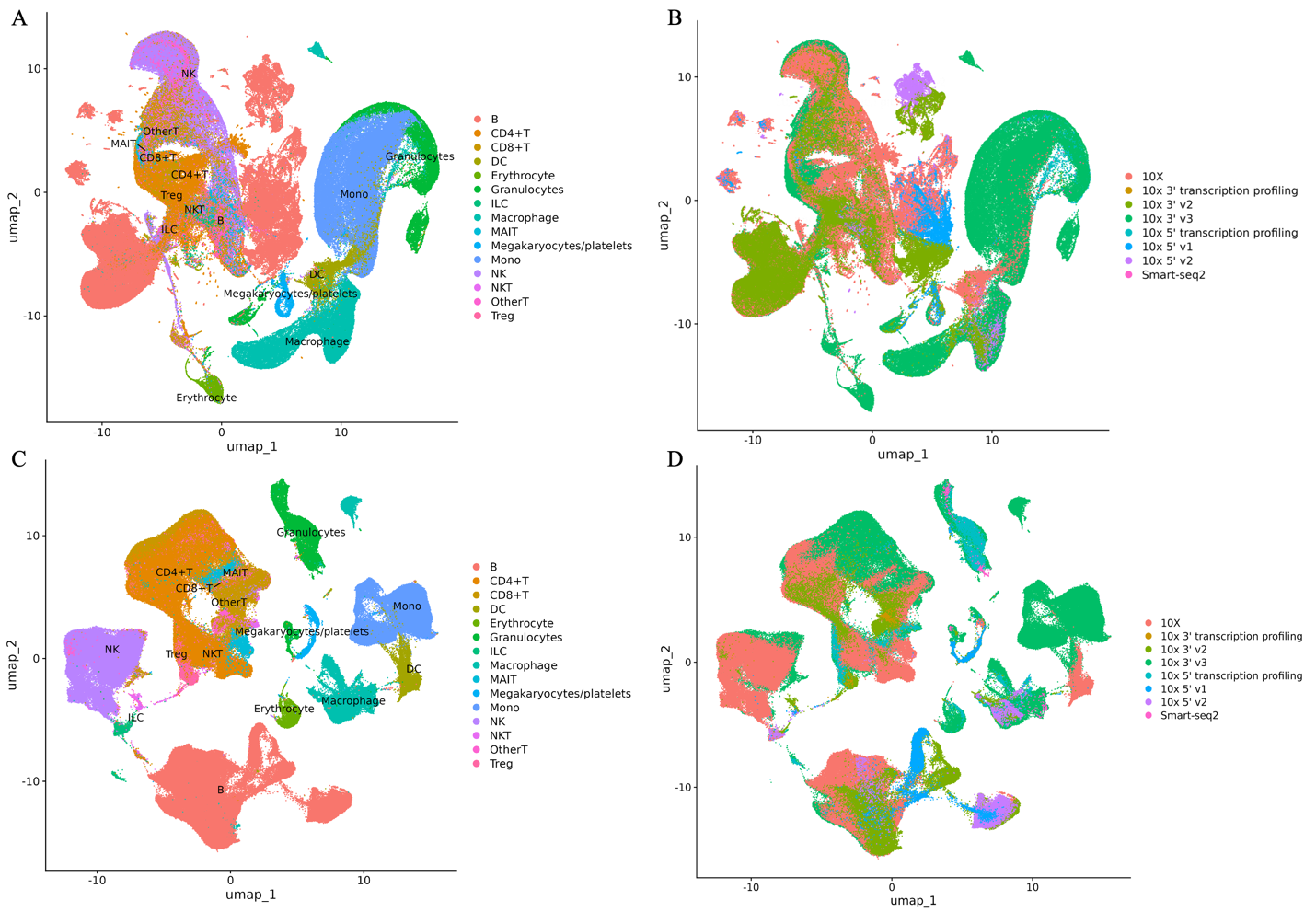


**Supplementary_Figure 8: Visualization of the batch effect correction results by STACAS integration on reference datasets used in scHDeepInsight. (A, B)** UMAP embeddings before batch effect correction, colored by cell type (A) and sequencing protocol (B). Pronounced batch effects are observed, with cells clustering primarily according to sequencing platform rather than biological identity, leading to poor separation of known immune cell types. **(C, D)** UMAP embeddings after STACAS integration, showing substantial removal of batch effects. Post-integration cells cluster primarily by biological identity (C) rather than technical platforms (D), demonstrating effective harmonization across datasets and enhanced biological coherence essential for accurate hierarchical annotation in scHDeepInsight.


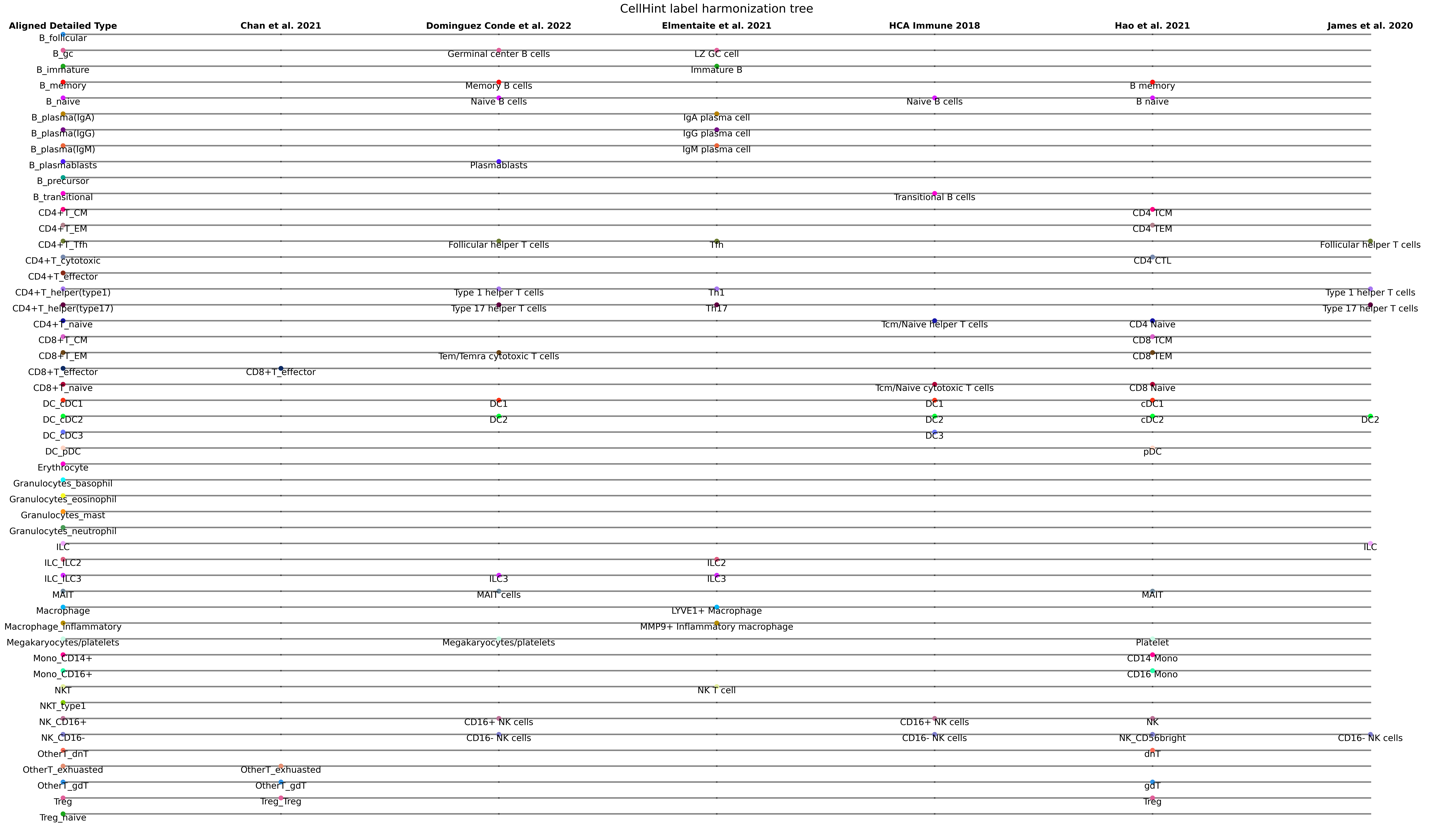


**Supplementary_Figure 9-1: Harmonization of cell type labels across six datasets using CellHint (part 1).** CellHint-based label harmonization for six immune-focused scRNA-seq datasets: Chan et al. 2021, Dominguez Conde et al. 2022, Elmentaite et al. 2021, HCA Immune 2018, Hao et al. 2021, and James et al. 2020. Rows represent harmonized immune cell subtypes used by scHDeepInsight, while each column shows the original cell labels from one dataset. Harmonization was performed using CellHint, which aligns diverse cell type annotations by leveraging transcriptomic similarity and a structured immune taxonomy. Additional datasets are shown in Supplementary_Figure 9-2.


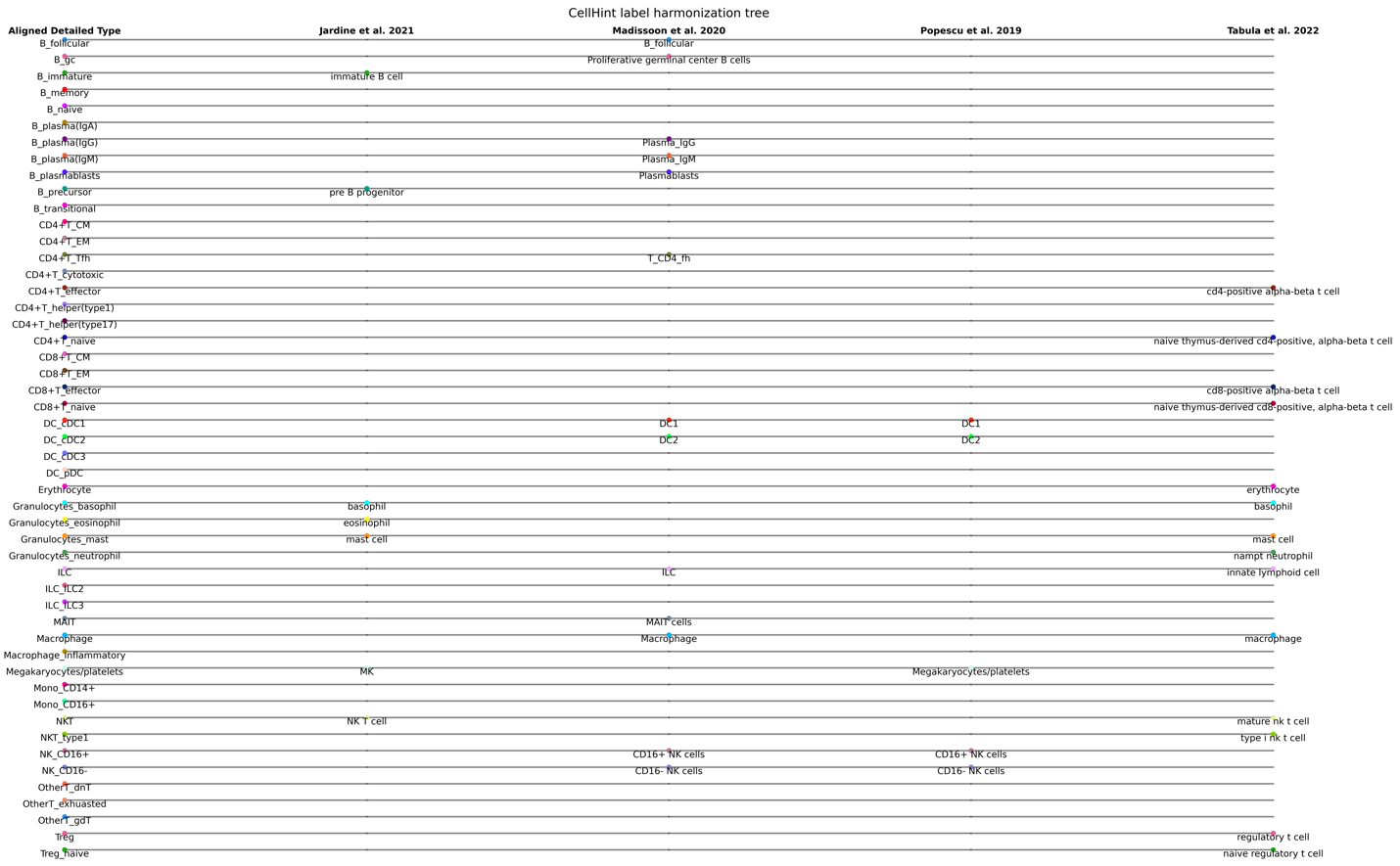


**Supplementary_Figure 9-2: Harmonization of cell type labels across six datasets using CellHint (part 2).** CellHint-based mapping of immune cell annotations for four datasets: Jardine et al. 2021, Madissoon et al. 2020, Popescu et al. 2019, and Tabula Sapiens 2022. Each column corresponds to a dataset's original cell type labels, and rows represent the standardized immune cell subtypes used in the scHDeepInsight reference atlas. Together with Supplementary_Figure 9-1, this harmonization ensures consistent and biologically meaningful subtype annotations across a diverse collection of scRNA-seq datasets


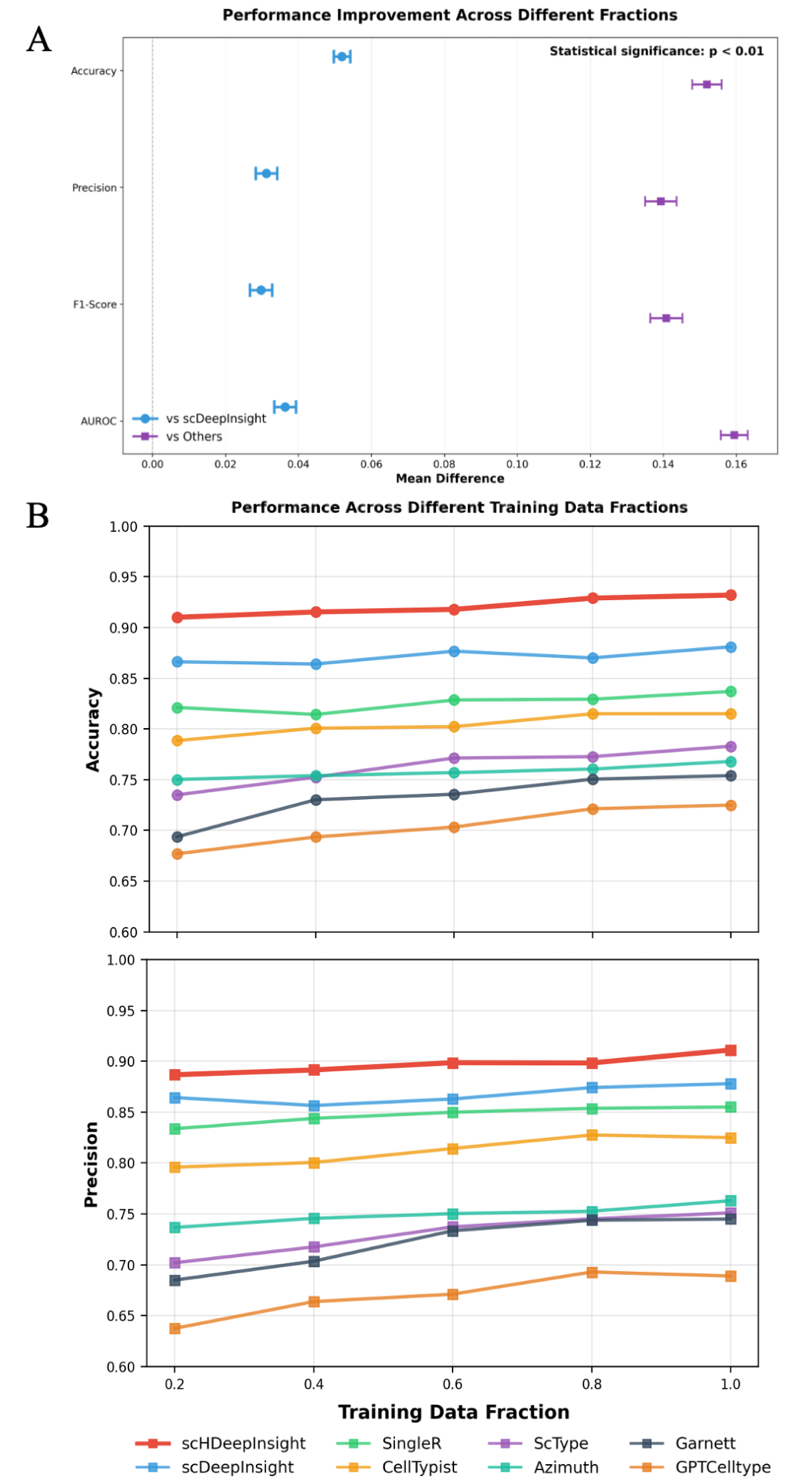


**Supplementary_Figure 10:** Statistical validation of scHDeepInsight performance improvements. **(A)** Mean performance differences with 99% confidence intervals across four metrics. Blue circles: vs scDeepInsight; purple squares: vs other methods. All comparisons significant at p < 0.01 (Wilcoxon signed-rank test, n=7 datasets). **(B)** Performance curves across training data fractions (20%-100%) showing accuracy (top) and precision (bottom) for all methods across seven datasets.


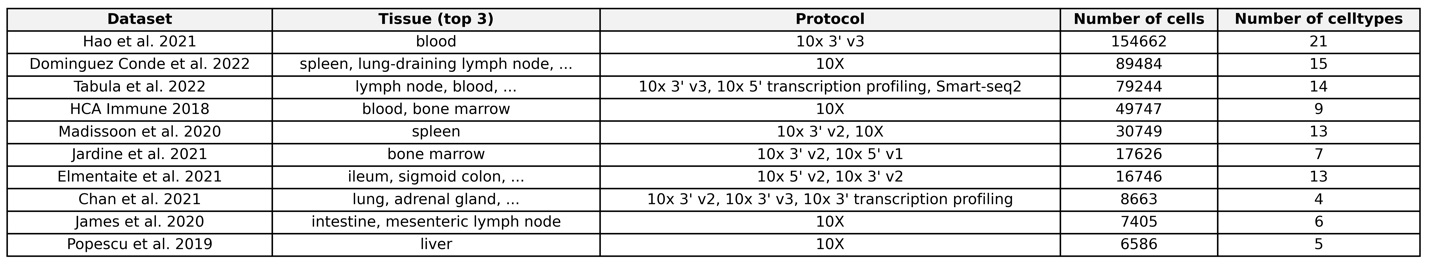


**Supplementary_Table 1: Summary of datasets used for constructing the integrated reference atlas utilized in scHDeepInsight.** The datasets included in the integrated reference, highlighting the tissues examined, sequencing protocols employed, the total number of cells analyzed, and the number of distinct immune cell types identified. The integration encompasses diverse scRNA-seq platforms such as various versions of 10x Genomics and Smart-seq, enhancing the biological coverage and robustness of the reference atlas.

**
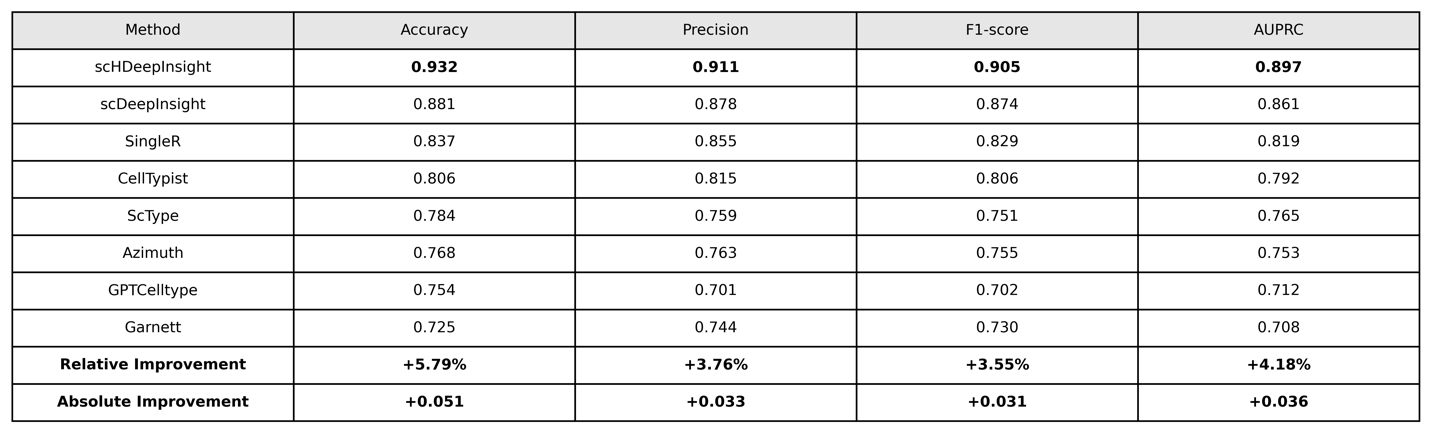
**

**Supplementary_Table 2: Benchmarking performance metrics comparing scHDeepInsight with other state-of-the-art immune cell annotation methods on seven independent query datasets.** The table summarizes average performance across four evaluation metrics: accuracy, precision, F1-score, and AUROC. Both relative (%) and absolute improvements achieved by scHDeepInsight over other seven methods (including scDeepInsight, SingleR, CellTypist, ScType, Azimuth, GPTCelltype, Garnett) are reported, highlighting the substantial enhancements provided by integrating hierarchical structure and adaptive focal loss.

**Supplementary_Note:**

**1. Data Preprocessing**

Rigorous data preprocessing is essential to ensure the quality and reliability of the scRNA-seq data prior to downstream analyses. The preprocessing workflow consists of three key steps: 1) Quality control (QC) filtering, and 2) Normalization and feature selection.

The QC filtering step removes low-quality cells and technical artifacts to ensure the dataset that accurately represents viable and healthy cells. First, cells were filtered based on specific quality metrics: Cells whose RNA feature counts and total RNA molecule counts fell within the 2nd to 99th percentiles were retained, removing potential doublets (excessively high counts) and low-quality cells (insufficient RNA content). Thereafter, cells with >15% mitochondrial gene expression were excluded, as elevated mitochondrial content often indicates cellular stress or apoptosis. These QC filtering criteria helped minimize technical artifacts while ensuring that the retained cells were biologically meaningful.

The normalization and feature selection steps were performed to correct for sequencing depth and technical variability, while preserving true biological variation. We applied scTransform, a regularized negative binomial regression model, to normalize gene expression and mitigate technical biases. As part of this process, scTransform [1] identifies highly variable genes (HVGs). In this study, we selected the top 5,000 HVGs based on the reference datasets for subsequent analyses. Focusing on these most informative features improved computational efficiency and helped ensure that the downstream hierarchical classification steps captured meaningful biological signals rather than technical noise.

**2. Batch Correction for Reference Atlas and Query Alignment**

Addressing batch effects is essential for constructing a cohesive reference atlas and enabling accurate annotation of new scRNA-seq datasets. We employed STACAS, a semi-supervised integration method because of its ability to preserve biological heterogeneity while effectively removing technical variation. Unlike some batch correction methods that may over-correct and merge biologically distinct populations, STACAS utilizes cell type annotations to guide integration when available, ensuring that true biological distinctions between immune cell types are maintained.

For reference dataset integration, STACAS leveraged known cell type annotations during batch correction, thereby ensuring the preservation of biologically meaningful distinctions between immune cell types. When applying the trained model to query datasets, STACAS was used in an unsupervised manner to map query cells into the reference expression space. This was achieved by leveraging a subset of 73,500 cells from the reference atlas as an intermediate structure, allowing query cells to be aligned onto the integrated reference without relying on prior labels. By ensuring that query cells project into a biologically meaningful embedding, this approach facilitates hierarchical classification while maintaining consistency between datasets.

As shown in Supplementary_Figure 7, batch correction strategy significantly improved the cohesion of cell type clusters (detailed visualization of protocol-specific batch effects before and after integration are shown in Supplementary_Figure 8).

Supplementary_Figure 8A illustrates the pre-integration state where batch effects cause artificial dispersion of similar cell types across the embedding space. In contrast, Supplementary_Figure 8B shows the post-STACAS integration, where cells of the same subtype form more coherent clusters while maintaining appropriate separation between biologically distinct populations. The improvement in silhouette score from 0.02 (pre-integration) to 0.24 (post-integration) quantitatively confirms STACAS's effectiveness in reducing technical variation while preserving biological identity, providing an ideal foundation for hierarchical classification approach.

**3. Evaluation of cell type annotations**

Multiple classification metrics were used to quantify the predictive performance, and cell type definitions were harmonized across datasets for consistent comparisons. This procedure facilitated an unbiased assessment of how the hierarchical framework and adaptive loss function contribute to improved cell type annotation relative to baseline methods.

**3.1 Cell Type Harmonization**

In scHDeepInsight, we recognized that different datasets often have varying granularity and nomenclature for cell type annotations. To address this variability, we employed both manual alignment and the CellHint [2] tool to ensure accurate cell type harmonization across datasets (the detailed harmonization tree is presented in Supplementary_Figure 9-1 and 9-2). CellHint operates by calculating transcriptomic similarities between cells and aligning them within a hierarchical graph, thereby harmonizing cell types at different levels of resolution. During the CellHint harmonization process, rare cell subtypes represented by fewer than 100 cells across all datasets were identified, and those lacking sufficient transcriptomic coherence or clear biological relationships were excluded from the training set to prevent overfitting. This process allowed us to maintain consistency in cell type definitions while also revealing underexplored relationships and potential novel cell types.

**3.2 Accuracy, Precision, F1-Score, and AUPRC Comparison**

The evaluation metrics used to measure performance included accuracy, precision, F1-score, and the Area Under the Precision–Recall Curve (AUPRC), which are standard metrics for assessing the quality of classification models.

Accuracy is calculated as follows:

$$Accuracy= \frac{TP+TN}{TP+TN+FP+FN} (1)$$

where $TP$, $TN$, $FP$, and $FN$ represent true positives, true negatives, false positives, and false negatives, respectively. scHDeepInsight achieved an average accuracy of 0.94 across all datasets.

Precision reflects the model's ability to correctly identify true positive instances out of all positive predictions. It is defined by the equation:

$$Precision= \frac{TP}{TP+FP} (2)$$

F1-Score combines precision and recall into a single metric:

$$F1-Score= 2*\frac{Precision*Recall}{Precision+Recall} (3)$$

Recall, also known as sensitivity or true positive rate, is calculated as:

$$Recall= \frac{TP}{TP+FN} (4)$$

In addition to accuracy, precision, and F1-score, our evaluation also includes AUPRC, which is particularly valuable in imbalanced datasets where certain immune cell subtypes appear at low frequency.

The AUPRC is computed as the integral of precision  $P$ with respect to recall  $R$:

$$AUPRC= \int_{0}^{1} P\left( R \right)dR (5)$$

Compared with the other metrics, the AUPRC more accurately reflects how well rare cell types are detected, offering a more nuanced assessment of classifier performance.

**3.3 Statistical Validation of Performance Improvements**

To provide robust statistical support for the performance comparisons presented in the main manuscript, we conducted comprehensive statistical validation across multiple experimental conditions. We evaluated each method across seven independent benchmark datasets using multiple training data fractions (20%, 40%, 60%, 80%, 100%) with 10 random sampling iterations per fraction. For each dataset, we calculated the mean performance difference across all experimental conditions, then performed Wilcoxon signed-rank tests on these seven dataset-level differences.

The statistical analysis confirms statistically significant improvements (p < 0.01) for scHDeepInsight compared to both scDeepInsight and other methods across all evaluation metrics (Supplementary_Figure 10A). When compared to scDeepInsight, scHDeepInsight achieved mean improvements of 5.2 percentage points in accuracy (±0.3), 3.2 percentage points in precision (±0.2), 3.0 percentage points in F1-score (±0.2), and 3.8 percentage points in AUPRC (±0.2). Comparisons with other state-of-the-art methods revealed more substantial improvements: 15.2 percentage points in accuracy (±0.4), 14.2 percentage points in precision (±0.4), 13.8 percentage points in F1-score (±0.3), and 15.8 percentage points in AUPRC (±0.4).

The performance trends across different training data fractions (Supplementary_Figure 10B) demonstrate scHDeepInsight's robustness under varying data availability conditions. ScHDeepInsight maintains consistently high performance even with limited training data (20% fraction), whereas competing methods exhibit more pronounced performance degradation under data-constrained scenarios.

**3.4 Rare Subtype Handling and Overfitting Prevention**

**3.4.1 Stratified Data Preparation**

To maintain representative coverage of all immune subtypes, dataset splitting employed subtype-level stratification rather than random or base-type-based approaches. Training and validation sets preserved proportional distributions across the full spectrum of abundant and rare populations within each immune lineage, ensuring adequate samples for both learning and assessment of underrepresented cell types.

**3.4.2 Hierarchical Feature Learning Benefits**

Rare subtypes with limited training data leverage lineage-specific feature representations learned from abundant populations within the same immune family through the two-stage classification architecture. Common features are established at the base-type level before fine-grained subtype distinction, allowing rare populations to benefit from larger sample sizes of related cell types and reducing effective sample size requirements for stable classification.

**3.4.3 Independent Dataset Validation Strategy**

Seven independent datasets from distinct studies, tissue types, and experimental protocols served as the validation framework for inter-dataset performance assessment. This design tests model generalizability against completely unseen biological contexts and experimental conditions, providing stringent assessment of rare subtype performance across diverse research environments.

**4. Overview of Cell Type Annotation Methods Used in Benchmarking**

**4.1 SingleR**

SingleR leverages a reference-based approach for cell type annotation, particularly well-suited for scRNA-seq datasets. The method involves a systematic comparison of gene expression profiles between individual cells in a query dataset and a pre-defined reference composed of annotated cell types. By computing similarity metrics, SingleR assigns cell types based on the closest match found in the reference. This approach is inherently flexible, as it does not require complex model training, relying instead on the quality and comprehensiveness of the reference dataset. Its application is most effective when there is confidence in the reference data's accuracy and relevance to the query dataset.

**4.2 CellTypist**

CellTypist addresses the need for rapid and scalable cell type annotation in the context of large scRNA-seq datasets. Unlike methods that require extensive parameter tuning, CellTypist utilizes pre-trained machine learning models, enabling it to process vast datasets efficiently. These models, trained on diverse cell types, can quickly classify cells based on gene expression profiles, providing consistent results across various tissues. CellTypist’s design caters to high-throughput scenarios, offering a streamlined solution for cell type identification with minimal user input, which is particularly advantageous in large-scale studies.

**4.3 Azimuth**

In contrast to methods that rely purely on reference-based or marker-based approaches, Azimuth stands out by employing transfer learning to enhance cell type annotation in scRNA-seq data. Azimuth projects query datasets into a shared low-dimensional space alongside a pre-annotated reference atlas. This alignment allows for the transfer of cell type annotations from the reference to the query, facilitating accurate classification even in datasets with significant batch effects or technical variations. Azimuth's strength lies in its ability to maintain high annotation accuracy across datasets that differ from the reference, making it a robust tool for integrative analyses.

**4.4 Garnett**

Garnett offers a unique marker-based approach to cell type annotation, particularly valuable when users possess detailed knowledge of marker genes specific to their study. Instead of relying on pre-existing models, Garnett allows users to construct their own hierarchical classifiers based on known markers. These classifiers are trained to recognize specific cell types within the query dataset, making Garnett highly customizable. This method is particularly effective in studies where researchers aim to identify or confirm the presence of specific cell populations based on well-characterized genetic markers.

**4.5 scType**

scType bridges the gap between marker-based and reference-based cell type annotation by integrating both strategies within a single framework. This hybrid approach enables scType to identify cell types using predefined marker genes while simultaneously validating these assignments against a reference dataset. The dual reliance on marker information and reference data allows scType to offer a balanced annotation solution, adaptable to various scRNA-seq datasets. This versatility makes it particularly suitable for studies where comprehensive reference data are available, but there is also a need to incorporate specific marker-based insights.

**4.6 GPTCellType**

GPTCellType takes a novel approach to cell type annotation by applying deep learning techniques, specifically generative pre-trained transformers (GPT), to scRNA-seq data. Drawing inspiration from natural language processing, GPTCellType interprets gene expression profiles as sequences, using a model trained on extensive scRNA-seq data to predict cell types. This method excels in capturing intricate relationships within the data, making it a powerful tool for identifying cell types in datasets characterized by high cellular diversity. GPTCellType’s deep learning foundation allows it to handle complex datasets that might challenge traditional annotation methods.

**References**

1. Hafemeister C, Satija R. Normalization and variance stabilization of single-cell RNA-seq data using regularized negative binomial regression. Genome Biology 2019; 20:296

2. Xu C, Prete M, Webb S, et al. Automatic cell-type harmonization and integration across Human Cell Atlas datasets. Cell 2023; 186:5876-5891.e20
